# ^99m^Tc/^90^Y radiolabeled biodegradable gel microspheres for lung shutting fraction assessment and radioembolization in hepatocellular carcinoma theranostics

**DOI:** 10.1016/j.mtbio.2024.101367

**Published:** 2024-11-27

**Authors:** Yi Dong, Lingling Yin, Jintao Huang, Di Hu, Jing Sun, Zhe Zhang, Zhihao Li, Bin-Yan Zhong, Ran Zhu, Guanglin Wang

**Affiliations:** a State Key Laboratory of Radiation Medicine and Protection, School of Radiation Medicine and Protection, Collaborative Innovation Center of Radiation Medicine of Jiangsu Higher Education Institutions, Soochow University, Suzhou, 215123, China; b Department of Interventional Radiology, The First Affiliated Hospital of Soochow University, Suzhou, 215006, China

**Keywords:** Hepatocellular carcinoma, Transarterial radioembolization, Radioactive microspheres, Lung shunt fraction assessment, Biodegradable microspheres

## Abstract

Transarterial radioembolization (TARE) is a well-established clinical therapy for the treatment of patients with intermediate to advanced hepatocellular carcinoma (HCC) or those who are ineligible for radical treatment. However, commercialized radioactive microspheres still have some issues, such as high density, complicated preparation, non-biodegradability. Furthermore, the use of different radioactive microspheres during TARE and lung shunt fraction assessment has led to inconsistencies in biodistribution in certain cases. This study employed biodegradable hyaluronic acid (HA) as the backbone and modified with bisphosphonate and methacrylic acid to prepare biodegradable gel microspheres (HAMS) using the water-in-oil emulsification and photo-crosslinking for labeling the diagnostic radionuclide of ^99m^Tc and therapeutic radionuclide of ^90^Y. Both ^99m^Tc radiolabeled HAMS (^99m^Tc-HAMS) and radiolabeled ^90^Y-HAMS (^90^Y-HAMS) were highly efficient in radiolabeling and exhibited excellent radiostability *in vitro* and *in vivo*. ^99m^Tc-HAMS are highly effective in assessing the LSF, while ^90^Y-HAMS, administered though TARE, are effective in inhibiting the growth of in situ HCC without any side effects. Both ^99m^Tc-HAMS and ^90^Y-HAMS have promising clinical applications in HCC theranostics.

## Graphical abstract

**Figure:**
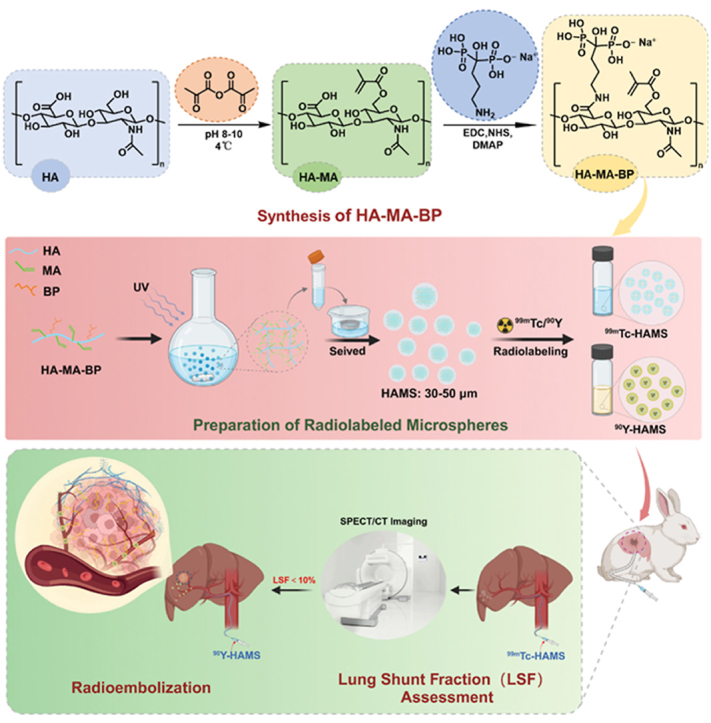

## 1 Introduction

Hepatocellular carcinoma (HCC) is a common and life-threatening disease that has a significant impact on public health and causes a substantial economic burden [1]. The mainstay approaches for HCC include resection, transplantation, percutaneous ablation, transarterial therapies, and systemic therapies [[2], [3], [4]]. Transarterial radioembolization (TARE) is offered for patients with intermediate-stage HCC and for very early and early-stage HCC patients in whom the former treatment options are not feasible [[5], [6], [7], [8]]. It involves delivering radioactive microspheres to the liver tumor through the hepatic artery, which releases radiation from radionuclides to kill the tumor cells [[9], [10], [11]]. Furthermore, TARE in conjunction with other modalities, such as photothermal therapy, demonstrates considerable inhibitory effects on hepatocellular carcinoma and represents a potentially exploitable treatment modality [12,13].

Commercial microspheres currently available in the market include TheraSphere™ (^90^Y-labeled glass microspheres manufactured by Boston Scientific), USA, SIR-Spheres® (^90^Y-labeled resin microspheres) manufactured by Sirtex Medical Ltd., Australia [14], and QuiremSpheres® (^166^Ho-PLA microspheres) manufactured by Quirem Medical B.V., The Netherlands [15,16]. Glass microspheres, due to their high density, can be challenging to formulate into suspensions [17]. However, they are non-biodegradable and can lead to uneven distribution within the tumor, resulting in permanent retention in the body. Resin microspheres have a low specific activity and a certain radionuclide leaching rate. Reactor activation is necessary for the production of ^166^Ho microspheres [18]. Long-time neutron activation can cause PLA microspheres to lose their spherical shape, rendering them unsuitable for interventional drug delivery.

Prior to TARE, it is mandatory to perform lung shunt fraction (LSF) assessment [19]. The imaging agent used for this purpose is ^99m^Tc radiolabeled micro aggregate albumin (^99m^Tc-MAA), which consists of micro albumin particles ranging from 10 to 100 μm [20]. However, due to the large size distribution of ^99m^Tc-MAA, it is not an accurate method to assess LSF in vessels affected by HCC [[21], [22]], [[21], [22]] which is crucial in determining the patient's suitability for TARE. Therefore, a biodegradable microsphere that can be used for both LSF assessment and TARE is desirable.

Hyaluronic acid (HA), also known as hyaluronan, is a linear glycine-glucosaminoglycan composed of N-acetyl D-glucosamine and D-glucuronic acid [23]. It is a high molecular weight acidic mucopolysaccharide. In the skin, HA accounts for over 50 % of the total HA in the body [24]. HA possesses unique viscoelasticity, excellent biocompatibility, and non-immunogenicity [25,26]. In addition, HA is a biodegradable material that can freely diffuse and degrade between tissues. Hyaluronidase HYALs are present in the liver, where they cleave hyaluronic acid into medium-sized fragments. Subsequently, these fragments are then internalized and degraded to monosaccharides by a combination of lysosomal hyaluronidases and exoglycosidases [[27], [28], [29], [30]]. It is widely used in the clinical setting as a key biomaterial due to its excellent biocompatibility [31]. Nano and micron particles made from HA are commonly used as a carrier for controlled release drug delivery and local radiotherapy due to their excellent properties [32]. For example, S. Kim. et al. synthesized a locally injectable ^131^I-labeled biodegradable HAMA microgel that showed great local retention and high biocompatibility [33]. Moreover, gel microspheres made from HA have a smooth surface, uniform size, perfect sphericity, and varying elasticity, making it an ideal material for interventional embolization [34].

**Fig. 1:**
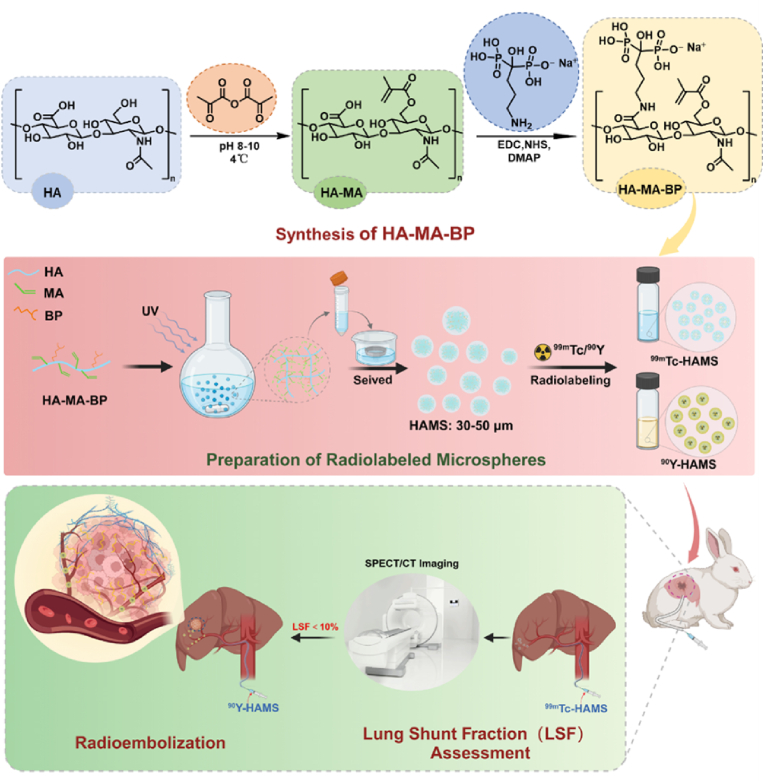
Schematic drawing of preparation of ^99m^Tc-HAMS and ^90^Y-HAMS, ^99m^Tc-HAMS was used for assessment of LSF before radioembolization and ^90^Y-HAMS for TARE of HCC.

This study describes the development of biodegradable gel microspheres (HAMS) created from HA derivatives that have been modified with methacrylic (MA) and bisphosphonate (BP) groups. The HAMS were prepared using water-in-oil emulsification and photo-crosslinking [35]. They contain bisphosphonate that can be labeled with diagnostic radionuclide of ^99m^Tc (γ, T_1/2_ = 6.0 h) and therapeutic radionuclide of ^90^Y (β, T_1/2_ = 2.64 days) through metal complexation. The ^99m^Tc-HAMS were used for preoperative LSF assessment of TARE by SPECT/CT imaging, while ^90^Y-HAMS were used for TARE in rabbits with in situ HCC (Fig. 1). The study found that ^99m^Tc-HAMS can be used to assess LSF and ^90^Y-HAMS can be used to inhibit HCC growth without significant side effects.

## 2 Experiment

Na^99m^TcO_4_ and ^99m^Tc-MAA were purchased from Shanghai GMS Pharmaceutical Co., Ltd. while ^90^YCl_3_ was obtained from Chengdu New Radiomedicine Technology Co., Ltd. All other chemicals were received and used commercially. The molecular weight of HA was 20–40 kDa.

### 2.1 Synthesis and characterization of HA derivatives and microspheres

4 mL of MA were added dropwise to 100 mL of a 2 % aqueous hyaluronic acid solution. The pH was then adjusted to 8–9 with 5 M NaOH [33]. The reaction was carried out in ice water for 24 h. Dialysis was performed in ultrapure water using dialysis bags (MW 8000–14000) for 3 days. After freeze-drying, the white spongy solid of HA-MA was characterized using NMR in deuterium water. The reaction mixture was prepared by adding 3.9 g of dicyclohexylcarbodiimide to 40 mL of 5 % aqueous HA-MA solution and incubating it for 30 min at 37 °C. Next, 4.8 g (41.6 mmol) of N-hydroxysuccinimide was added, and the reaction was maintained for 6 h. Separately, 3.4 g (10.4 mmol) of alendronate trihydrate and 1.3 g (10.4 mmol) of 4-dimethylaminopyridine were dissolved in 15 mL of ultrapure water and added dropwise to the above solution for 48 h. After freeze-drying, ^1^H NMR hydrogen and ^31^P NMR spectra were performed on HA-MA-BP in deuterium water.

200 mg of HA-MA-BP was added to 2 mL of a 0.5 % (w/v) LAP solution. Next, 40 mL of n-octanol was mixed with a 10 % volume of Spectra 80 (Sp-80) to make a 10 % (v/v) n-octanol solution. The oil phase was stirred while the aqueous phase was slowly added. The reaction solution was then irradiated with UV light at a wavelength of 365 nm for 10 min to solidify the small droplets of the aqueous phase that were dispersed in the oil phase, resulting in the formation of microspheres. The microspheres were obtained through centrifugation and separation, and different sizes were achieved by a standard sieve. The morphology and size of the gel microspheres were measured by optical microscopy (Olympus, IX73, Japan) and scanning electron microscopy (Zeiss, EVO 18, Germany), respectively. For purpose of the evaluation of the deformability of the HAMS, compression deformation tests were performed on the microspheres under different physical pressures, and the morphological changes of the microspheres in aqueous solution were recorded under light microscopy before and after compression.

### 2.2 Radiolabeling and *in vitro* radiostability

1 mCi Na^99m^TcO_4_ solution (approximately 100 μL) was mixed with 20 μL of SnCl_2_ solution (1 mg/mL, pH = 1) for 5 min. Next, 100 μL of microsphere solution was added, and the reaction mixture was placed on a thermostatic homogenizer at 37 °C and 1500 rpm for 30 min. After the reaction, the microsphere solution was centrifuged at 3000 rpm for 3 min and washed five times with deionized water to remove any free radionuclides. The resulting product was ^99m^Tc-labeled hyaluronic acid gel microspheres (^99m^Tc-HAMS). For the ^90^Y radiolabeling process, 1 mCi of ^90^Y was added to 5 mg of hyaluronic acid microspheres in 100 μL of deionized water. The mixture was then reacted at 50 °C for 50 min while shaking. After the reaction, the solution was washed five times with deionized water to remove any free radionuclides. This resulted in the production of ^90^Y-labeled hyaluronic acid gel microspheres (^90^Y-HAMS). The radiolabeling efficiency was determined by dividing the activity of ^90^Y-HAMS or ^99m^Tc-HAMS by the added activity. The activity was measured using a dose calibrator (Capintec, CRC-25R, USA).

200 μCi of ^99m^Tc-HAMS or ^90^Y-HAMS was added to 1 mL of PBS or 10 % FBS at room temperature. The activity of free radionuclides was measured using a gamma counter after centrifugation at various incubation times.

### 2.3 *In vitro* degradation assay

To assess the degradation of HAMS in the presence of hyaluronidase, the microspheres were dispersed in 1 mL of hyaluronidase PBS solution containing 600 IU/mL or 1200 IU/mL at 37 °C. Every three days, the HAMS were collected, centrifuged at 3000 rpm for 3 min, and the resulting precipitate was collected, freeze-dried, and weighed. Furthermore, a fresh hyaluronidase PBS solution was replaced daily. The microspheres degradation rate was determined by calculating the mass ratio of the remaining to the initial mass of HAMS. In addition, SEM was employed to observe alterations in microsphere morphology (Zeiss, EVO 18, Germany).

### 2.4 Cytotoxicity and immunofluorescence cell assay

The cytotoxicity of microspheres was investigated by incubating various concentrations or activity of HAMS, ^99m^Tc-HAMS or ^90^Y-HAMS ranging from 0 to 1000 g/mL or 0–50 μCi incubated in HUVEC, McA-RH7777, and VX2 cells at 37 °C 5 % CO_2_ atmospheres for 24 h. Residual microspheres and culture medium were then removed with PBS. Next, 100 μL of DMEM and 10 μL of CCK-8 were added to the cells and cultured for 1 h. The absorbance of each well at 450 nm was measured using a multifunctional microplate reader (Thermo Scientific, Varioskan Flash, Japan).

VX2 cells were cultured on cell slides and incubated with HAMS, ^99m^Tc-HAMS, and ^90^Y-HAMS for 24 h. The cells were washed with PBS three times after 15 min of 4 % paraformaldehyde fixation. Following this, 0.5 % of Triton X-100 was added to the cells to rupture the cell membrane for 15 min. The cells were rinsed twice with PBS and then treated with 5 % bovine serum albumin in Tris-buffered saline solution for 1 h at 37 °C. Then, the cells were incubated with anti-histone γH2AX mouse monoclonal antibodies incubated for 2 h at 37 °C. After washing with PBS five times, the cells were then exposed to rabbit anti-mouse secondary antibody for 1 h at 37 °C in the dark. Finally, the nuclei were stained with Hoechst (20 μL, 5 mg/mL) for 5 min. Fluorescence imaging was performed at the end using a confocal laser scanning microscope (Olympus FV1200, Japan). The resulting fluorescence images were analyzed using ImageJ software.

### 2.5 *In vivo* imaging

To assess the imaging capability of ^99m^Tc-HAMS, mice were intravenously injected with ^99m^Tc-HAMS (500 μCi) and ^99m^Tc-MAA (500 μCi) via the tail vein. The N1S1 tumor model was established as reported in the literature [36]. Rats with in situ N1S1 tumors were embolized with ^99m^Tc-HAMS (500 μCi) via the hepatic artery. The biodistribution of radiolabeled microspheres was monitored using microSPECT/CT. The SPECT scan rate was set to 15 min per frame, and full-angle scanning (615 mA, 55 kV) was used for the CT scans. The PMOD software version 3.602 was used to analyze the SPECT images. Following the SPECT scan, the mice were sacrificed, and their major organs were harvested and weighed. The activity of each organ was measured using a gamma counter (Capintec, CRC-25R, USA).

### 2.6 Evaluation of vascular embolization

A total of 200 μL of HAMS (10 mg/mL) were injected into the middle artery of the rabbit's ear, and pressure was applied to stop bleeding. The morphological changes in the rabbit ear were continuously observed in order to assess the effect of microsphere embolization.

### 2.7 Antitumor therapy

To evaluate the tumor inhibition of ^90^Y-HAMS, white New Zeeland rabbits (weighing 3–3.5 kg) with VX2 in situ liver cancer model were used. Rabbits with tumor volumes ranging from 350 mm^3^ to 500 mm^3^ were divided into three groups: saline (control group), HAMS group, and ^90^Y-HAMS group. Saline (0.2 mL), HAMS (0.2 mL, 5 mg/mL), and ^90^Y-HAMS (0.2 mL, 5 mg/mL, 200 μCi) were embolized via transarterial administration. The tumor volume was monitored by enhanced CT on days 0, 7 and 14, and MRI on days 0 and 14. Liver enzymes, including alanine aminotransferase (ALT), alkaline phosphatase (ALP), aspartate aminotransferase (AST), creatinine (CREA), glutamylglutamyltransferase (GGT), and urea (UREA), were measured by drawing dorsal auricular arterial blood on days 0, 1, 3, 7, and 14 after embolization to evaluate liver and kidney function. Additionally, the activity of the blood was measured using a gamma counter. On day 14 after embolization, the rabbits were euthanized and major organs including the heart, liver, spleen, lungs, kidneys, and tumors, were collected for hematoxylin and eosin (H&E) staining. Tumor slices were also stained using terminal deoxynucleotidyl transferase dUTP nick-end labeling (TUNEL).

## 3 Results and discussion

### 3.1 Synthesis and characterization of radiolabeled microspheres

**Fig. 2:**
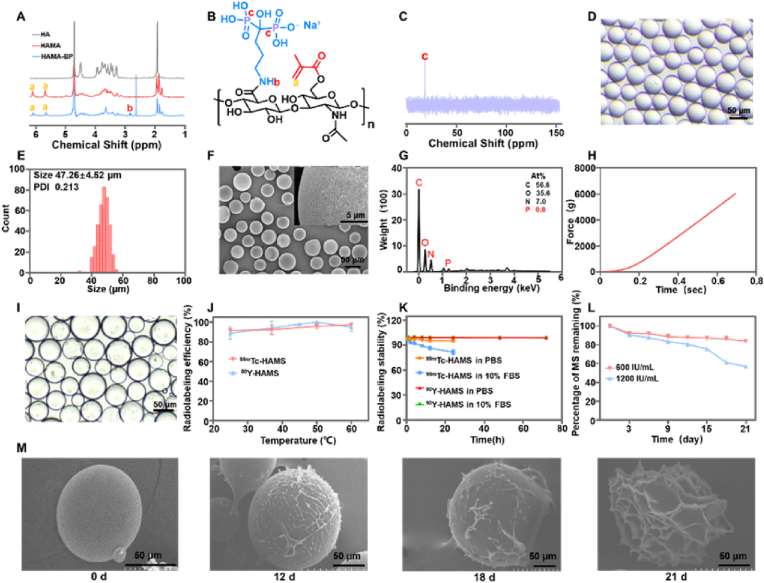
(A) The ^1^H NMR spectrum of HA, HA-MA and HA-MA-BP in deuterium water. (B) The chemical structure of HA-MA-BP. (C) The ^31^P NMR of HA-MA-BP in deuterium water. (D) Optical microscope image of suspensions of HAMS. (E) Histogram of HAMS analyzed from optical image. (F) SEM image of HAMS. (G) The energy spectrum of SEM of HAMS. (H) Pressure versus time curves for HAMS. (I) Optical image of microspheres following compression testing. (J) Radiolabeling efficiency of ^99m^Tc-HAMS and ^90^Y-HAMS incubated with various temperatures. (K) Radiostability of ^99m^Tc-HAMS and ^90^Y-HAMS in saline and 10 % FBS with various incubation times ranges from 0 h to 72 h, respectively. (L) The degradation rate curves of HAMS were generated on days 3, 6, 9, 12, 15, 18, and 21 after co-incubation with different concentrations of hyaluronidase. (M) SEM images of HAMS on days 0, 12, 18 and 21 following co-incubation with hyaluronidase (600 IU/mL).

To prepare the functional microspheres, HA was modified with MA and BP groups. MA was used for crosslinking during microsphere preparation, while BP was used for radiolabeling with ^99m^Tc and ^90^Y. The synthesis of HA-MA-BP was carried out in two steps. Firstly, methacrylic anhydride was used to esterify the hydroxyl group of HA. Secondly, BP was used to condense with the carboxyl group of HA-MA through an amide reaction (Fig. 1). Fig. 2A showed the ^1^H NMR spectrum of HA and HA derivatives, which revealed the characteristic peaks of MA with chemical shifts at 5.77 ppm and 6.20 ppm, and the characteristic peak of BP with a chemical shift at 2.95 ppm, indicating the successful synthesis of HA-MA-BP (Fig. 2B). The grafting rate was calculated to be 70.9 % according ^1^H NMR spectrum (Fig. S1). The successful synthesis of HA-MA-BP was also confirmed by the presence of phosphate elements in the ^31^P NMR (Fig. 2C). HAMS was prepared using the water-in-oil method and standard sieves to obtain an optimal size of 30–50 μm for radioembolization. Fig. S2 showed the graph image of HAMS powder in a vial, while the optical microscopy image demonstrated that it was spherical and of uniform size in deionized water. Upon analysis of the images, it was found that the size of HAMS was 47.26 ± 4.52 μm with a narrow polydispersity index of 0.21 (Fig. 2D and E). Microspheres with a size of 30–50 μm are the most suitable for use as TARE [37]. Additionally, SEM images revealed the spherical shape and gel structure of HAMS, with surface holes clearly visible (Fig. 2F). The energy spectrum of SEM, as shown in Fig. 2G, demonstrated the presence of phosphate elements in HAMS, which further suggested that the successful synthesis of HA-MA-BP. The microspheres exhibited minimal deformation following compression tests at various pressures, as illustrated in Fig. 2H–and I.

The bisphosphate group complexes with metal ions and can be used to label metal radionuclides. The radiolabeling efficiency of ^99m^Tc-HAMS was 97.3 ± 0.8 % at 60 °C with a 30-min reaction time, which increased with higher reaction temperatures (Fig. 2J). Similarly, the radiolabeling efficiency of ^90^Y-HAMS reached 100 % at 50 °C for a 50-min reaction time (Fig. 2J). The specific activity of ^90^Y-HAMS was 429.2 Bq per microspheres, which is considerably higher than that of the resin microsphere (75 Bq per microspheres). After radiolabeling, the size and morphology of the microspheres remained unchanged (Fig. S3). The radiostability of ^99m^Tc-HAMS and ^90^Y-HAMS was investigated in PBS and 10 % FBS with varying incubation times at room temperature (Fig. 2K). The radiostability of ^99m^Tc-HAMS was 97.19 ± 0.23 % in PBS and 92.35 ± 1.88 % in 10 % FBS after 4 h of incubation, which is higher than 95 % in PBS and 80 % in 10 % FBS even after 24 h incubation. These results indicated that ^99m^Tc-HAMS have high radiostability. Similarly, the radiostability of ^90^Y-HAMS was 99.1 ± 0.21 % in PBS and 98 ± 0.15 % in 10 % FBS after 72 h of incubation higher than that of the resin microsphere (97 % in saline in 24 h) [38], suggesting that ^90^Y-HAMS also had excellent radiostability. Bisphosphate groups can effectively form stable complexes with metal ions [39]. The above results demonstrated that radiolabeled microspheres had excellent radiostability for further *in vivo* study.

### 3.2 Degradation study of HAMS

HA is a biodegradable polymer that can be degraded in the presence of hyaluronidase [40,41]. The *in vitro* degradation of HAMS in the presence of hyaluronidase was illustrated in Fig. 2L. The rate of its degradation increased in direct proportion to the hyaluronidase concentration. Furthermore, the SEM images demonstrated that the integrity of HAMS was compromised over time (Fig. 2M), indicating that HAMS can be degraded by hyaluronidase. The graphs also indicated that the integrity of HAMS was not significantly disrupted within 18 days, which meets our requirement of no or very little degradation within a half-life of 4–5 of ^90^Y. Moreover, the concentration of the enzyme *in vivo* was found to be lower than that observed *in vitro* [42], resulting in slower degradation times. Consequently, it can be concluded that HAMS undergo degradation following radioactive decay.

### 3.3 Cytotoxicity

**Fig. 3:**
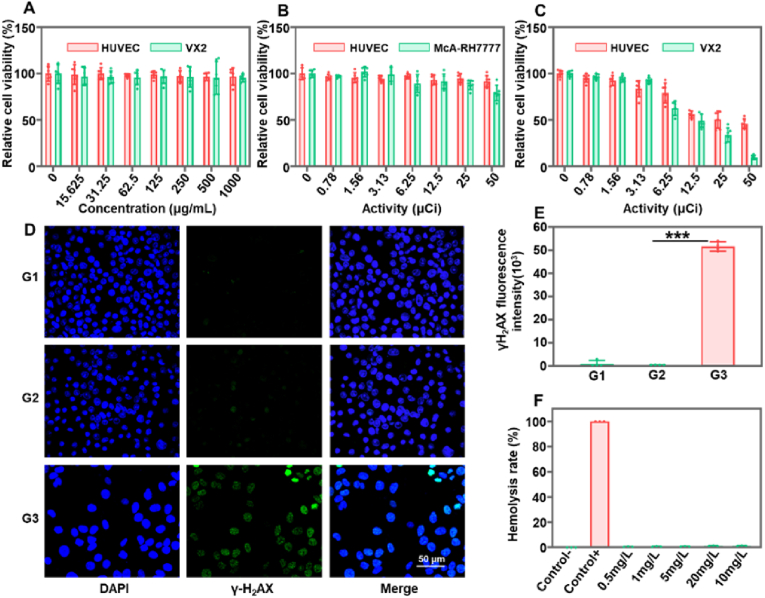
(A) Cell relative viability of HUVEC and VX2 cells after co-incubation with various concentrations of HAMS ranging from 0 to 1000 μg/mL. (B) Relative cell viability of HUVEC and McA-RH 7777 cells after co-incubation with different activities of ^99m^Tc-HAMS ranging from 0 to 50 μCi. (C) Relative cell viability of HUVEC and VX2 cells after co-incubation with different activities of ^90^Y-HAMS ranging from 0 to 50 μCi. (D) CLSM images of HUVEC and VX2 cells with different treatments (G1: saline, G2: ^99m^Tc-HAMS, G3: ^90^Y-HAMS). (E) Analyzing from CLSM images, γH_2_AX intensity of DNA damage with different treatments (G1: saline, G2: ^99m^Tc-HAMS, G3: ^90^Y-HAMS). (F) The percentage of hemolysis after co-incubation of diluted mouse blood with various concentrations of HAMS range from 0.5 mg/mL to 20 mg/mL at 37 °C. Hemolysis rate <2 % is considered nonhemolysis, 2%–5% is considered microhemolysis, and >5 % of the sample is considered hemolysis. *P* values were calculated by one-way ANOVA with Tukey multiple-comparison test, ∗∗∗*P* < 0.001.

Prior to further *in vivo* studies, the cytotoxicity of radiolabeled microspheres was evaluated using HUVEX, VX2, and McA-RH7777 cells. The cell viability of HUVEC and VX2 showed no significant changes with varying concentrations of HAMS from 0 to 1000 μm/mL (Fig. 3A). Additionally, no significant changes in cell viability were observed in HUVEC and McA-RH7777, even when the activity of ^99m^Tc-HAMS was increased to 50 μCi (Fig. 3B). The decay process of ^99m^Tc emits γ-rays, and the amount of γ-rays used for diagnosis is essentially nontoxic [43]. These results indicated that HAMS and ^99m^Tc-HAMS have good biocompatibility and are safe for further *in vivo* studies. The cell viability of HUVEC and VX2 cells significantly decreased with increased incubation activity of ^90^Y-HAMS (Fig. 3C). At 12.5 μCi incubation, 49.1 ± 6.9 % of VX2 cells survived, while only 9.5 ± 2.2 % survived at 50 μCi incubation, indicating that ^90^Y-HAMS can effectively kill VX2 tumor cells. ^90^Y releases high-energy electrons during decay, which can effectively kill cells. It is often used as a therapeutic radionuclide to treat tumors [44,45]. Additionally, an immunofluorescence cell assay was used to test for DNA damage. The results showed no obvious DNA damage in HUVEC cells treated with HAMS and ^99m^Tc-HAMS. However, the treatment with ^90^Y-HAMS resulted in significant DNA damage and lower VX2 density (Fig. 3D and E). The hemocompatibility of HAMS was also tested in blood, and no hemolysis was observed even at a concentration of 20 mg/mL (Fig. 3F and S4), further demonstrating the safety of HAMS. The cytotoxicity results indicated that ^99m^Tc-HAMS are safe for imaging, while ^90^Y-HAMS can effectively kill cancer cells.

### 3.4 *In vivo* imaging and biodistribution

**Fig. 4:**
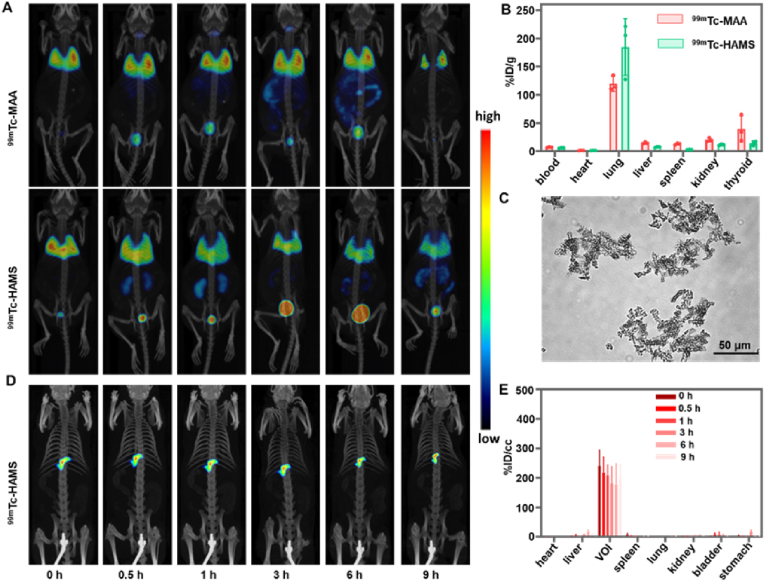
(A) SPECT/CT images of ^99m^Tc-MAA and ^99m^Tc-HAMS in mice intravenously injected via tail vein. (B) *In vivo* biodistribution of mice (n = 3) intravenously injected with ^99m^Tc-MAA and ^99m^Tc-HAMS at 9 h after injection. (C) Optical image of ^99m^Tc-MAA suspension. (D) SPECT/CT images of rat injected with ^99m^Tc-HAMS with hepatic artery injection. (E) The biodistribution of ^99m^Tc-HAMS after hepatic artery injection quantified from SPECT images.

Currently, ^99m^Tc is the primary radionuclide used in medical diagnostics, accounting for 80 % of diagnostic radionuclides worldwide due to its excellent nuclear properties, suitable half-life, and convenient availability [[46], [47], [48]]. To evaluate the imaging of LSF with ^99m^Tc-HAMS, mice were intravenously injected with ^99m^Tc-HAMS and ^99m^Tc-MAA through the tail vein and rats were injected with ^99m^Tc-HAMS through the hepatic artery and visualized with *micro*SPECT/CT. Following a tail vein injection, the microspheres were transported back to the right atrium via the inferior vena cava, where they were then distributed to the pulmonary capillaries via the left atrium. The *micro*SPECT images showed that the radioactive signals were primarily present in the lungs immediately after the injection of ^99m^Tc-HAMS and ^99m^Tc-MAA, and in the kidneys and bladder over time (Fig. 4A). Additionally, the signal of ^99m^Tc-MAA was detected in the thyroid gland. Nine hours after injection, the mice were euthanized, and their major organs were harvested for activity and mass measurements. The results indicated that ^99m^Tc-HAMS were more trapped in the lungs than in to other organs, which were significantly enriched than ^99m^Tc-MAA (184.7 ± 50.0 percentage of injection dose per gram (%ID/g) vs 119.8 ± 13.4 %ID/g) (Fig. 4B). The reason for this is likely since ^99m^Tc-HAMS were spheres that range from 30 to 50 μm in size, which was too large for them to pass through capillaries. As a result, they were trapped more by the lungs. On the other hand, the commercial ^99m^Tc-MAA particles had a larger size distribution and may not be well trapped in blood vessels (Fig. 4C). On the other hand, the use of same microspheres for both assessment and embolization may result in a more consistent distribution within the tumor. This suggests that ^99m^Tc-HAMS provided better imaging of lung capillary blockage. In addition, *micro*SPECT images of transhepatic arterial administration of ^99m^Tc-HAMS in rats showed that HAMS was effectively retained in the volume of interest (VOI) without significant changes over time (Fig. 4D). Quantitatively, ^99m^Tc-HAMS over 190 %ID/cc (percentage of injection dose per volume) remained in the VOI after 9 h and less than 3 %ID/cc in other organs (Fig. 4E). The above results demonstrated that ^99m^Tc-HAMS can be effectively used as a diagnostic radiopharmaceutical for evaluating LSF before TARE.

### 3.5 Evaluation of vascular embolization

**Fig. 5:**
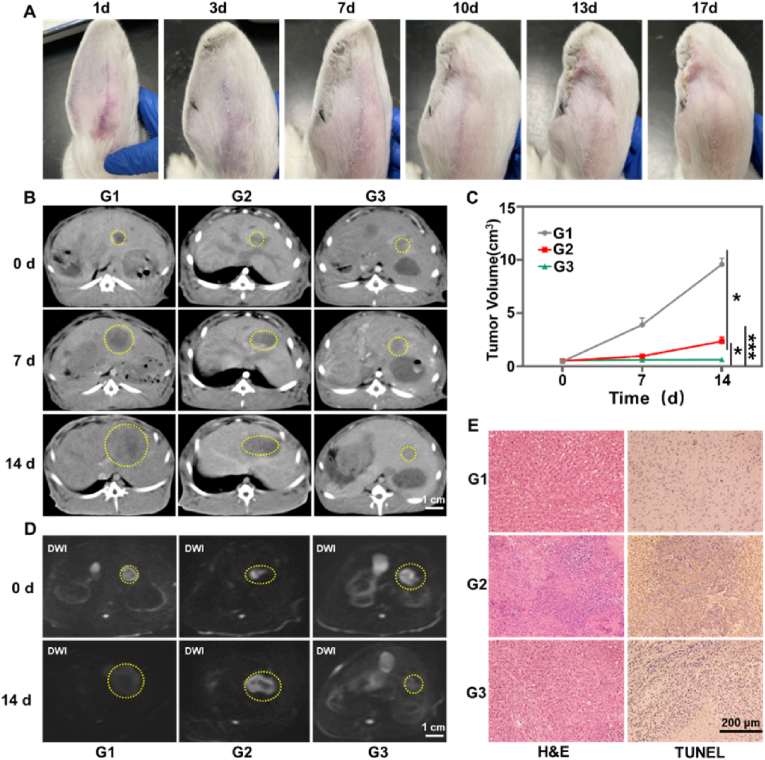
(A) The graphs of rabbit ear over time (1, 3, 7, 10, 13, and 17 days) following microspheres embolization. (B) The enhanced CT of rabbits treated with different treatments (G1: saline, G2: HAMS, G3: ^90^Y-HAMS) by embolization administration. The tumors were highlighted with yellow dashed circles. (C) The temporal tumor volume was recorded after different treatments (n = 3). (D) MRI images (DWI) of rabbits treated with different treatments (G1: saline, G2: HAMS, G3: ^90^Y-HAMS) by embolization administration. The tumors were highlighted with yellow dashed circles. (E) Macroscopic images of VX2 tumor slices obtained 14 days post-treatment and stained with H&E and TUNEL, respectively. *P* values were calculated by one-way ANOVA with Tukey multiple-comparison test, ∗*P* < 0.05, ∗∗*P* < 0.01, ∗∗∗*P* < 0.001.

Microspheres are typically endowed with some degree of embolic function [49]. To evaluate the embolic capacity of HAMS, an arterially injected rabbit ear model was employed. As shown in Fig. 5A, microspheres obstructed blood flow within the tiny blood vessels, resulting in the manifestation of ischemic necrosis at the ear margin. This indicated that HAMS were able to accurately enter the target site and produce an embolic at that site.

### 3.6 *In vivo* antitumor

**Fig. 6:**
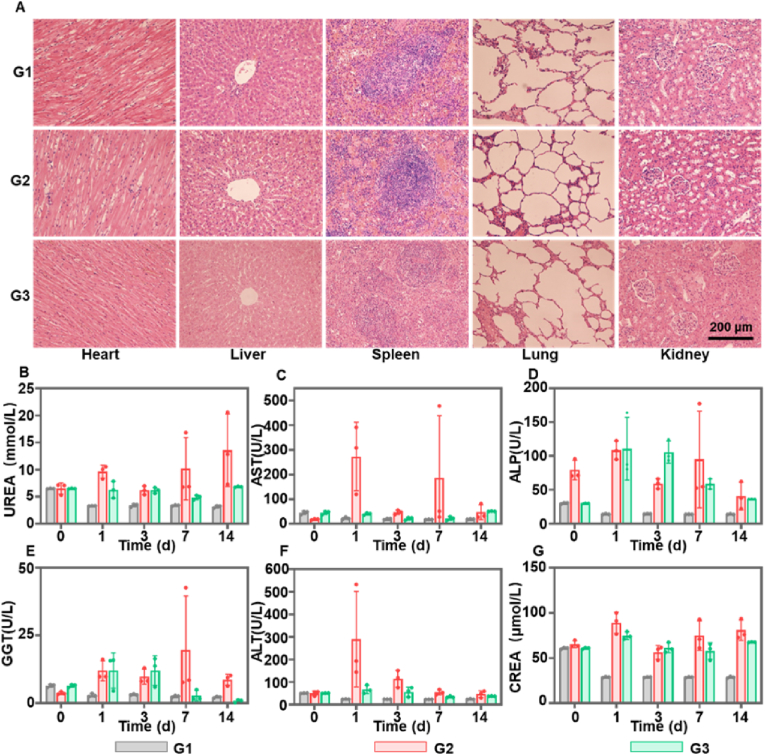
(A) Macroscopic H&E staining images of different organs of VX2 tumor-bearing rats harvested 14 days post-treatment (G1: saline, G2: HAMS, G3: ^90^Y-HAMS). (B–G) Expression levels of UREA, AST, ALP, GGT, ALT, and CREA on days 0, 3, 7, and 14 after treatment (G1: Saline; G2: HAMS; G3: ^90^Y-HAMS), respectively.

To evaluate the radioembolization capability of ^90^Y-HAMS, we used rabbit model with in situ VX2 tumor administered the treatment transarterial. The rabbits were divided into three groups, each consisting of three rabbits: saline group (200 μL), HAMS group (1 mg/200 μL), and ^90^Y-HAMS group (200 μCi, 1 mg/200 μL). CT scans were conducted at 0, 7, and 14 days after embolization, while MRI scans were conducted at 0 and 14 days after embolization. The CT and MRI images showed that there was a significantly reduction in tumor volume in the ^90^Y-HAMS group compared to the saline and HAMS groups (Fig. 5B–D and S5). On the fourteenth day of treatment, all rabbits were euthanized, and major organs and tumor tissues were taken for H&E staining. The tumor slices were then analyzed for terminal deoxynucleotidyl transferase dUTP nick labeling (TUNEL) staining. Fig. 5E demonstrates that tumors in the ^90^Y-HAMS treatment group exhibited significant cell necrosis and apoptosis, while major organs remained undamaged (Fig. 6A). In comparison to non-degradable microspheres, HAMS are biocompatible and have a low inflammatory potential, rendering them safe for use, particularly in patients with poor liver function. Meanwhile, HAMS can be degraded after a period of time, allowing for secondary embolization [[50], [51], [52]]. The liver enzymes were tested on days 0, 1, 3, 7, and 14 after embolization. The levels of AST, ALP, GGT, and ALT were elevated after embolization but returned to normal levels within a week (Fig. 6B–G), which is a typical symptom of embolization procedures [53]. Meanwhile, activity of blood and feces was monitored at 1, 3, 7, and 14 days after treatment. The levels of activity of ^90^Y in both blood and feces were similar to the background levels after embolization, proving further evidence of the high stability of ^90^Y-HAMS *in vivo*. These results demonstrated that ^90^Y-HAMS can effectively inhibit tumor growth with high safety.

## 4 Conclusion

In summary, novel microspheres radiolabeled with ^99m^Tc and ^90^Y have been developed for LSF evaluation and TARE of HCC. Both ^99m^Tc-HAMS and ^90^Y-HAMS are highly efficient in radiolabeling and exhibit excellent radiostability *in vitro* and *in vivo*. ^99m^Tc-HAMS demonstrated an excellent ability to assess LSF, while ^90^Y-HAMS, when administered through interventional embolization, effectively inhibited the growth of in situ HCC without any side effects. Both ^99m^Tc-HAMS and ^90^Y-HAMS show promising clinical theranostic applications.

## CRediT authorship contribution statement

**Yi Dong:** Writing – original draft, Investigation, Data curation, Conceptualization. **Lingling Yin:** Writing – original draft, Investigation, Data curation, Conceptualization. **Jintao Huang:** Methodology, Investigation. **Di Hu:** Methodology, Investigation. **Jing Sun:** Methodology, Formal analysis, Conceptualization. **Zhe Zhang:** Methodology, Investigation, Data curation, Conceptualization. **Zhihao Li:** Methodology, Investigation, Data curation. **Bin-Yan Zhong:** Writing – review & editing, Project administration, Funding acquisition, Conceptualization. **Ran Zhu:** Writing – review & editing, Resources, Project administration, Funding acquisition, Data curation, Conceptualization. **Guanglin Wang:** Writing – review & editing, Writing – original draft, Resources, Project administration, Investigation, Funding acquisition, Formal analysis, Data curation, Conceptualization.

## Ethical statement

All animal experiments were approved by the Animal Care and Use Committee of Soochow University, and all protocols of animal studies conformed to the Guide for the Care and Use of Laboratory Animals of Soochow University, P. R. China.

## Declaration of competing interest

The authors declare no competing financial interest.

## Data Availability

Data will be made available on request.
