# Supplementary material for: 99mTc/90Y radiolabeled biodegradable gel microspheres for lung shutting fraction assessment and radioembolization in hepatocellular carcinoma theranostics
Source: Mater Today Bio. 2024 Nov 27;29:101367. doi: 10.1016/j.mtbio.2024.101367 (PMC11647226; doi:10.1016/j.mtbio.2024.101367)
Supplement: Multimedia component 1 [file mmc1.docx]

**Supporting Information**

**^99m^Tc/^90^Y Radiolabeled Biodegradable Gel Microspheres for Lung Shutting Fraction Assessment and Radioembolization in Hepatocellular Carcinoma Theranostics**

Yi Dong^1, #^, Lingling Yin^1, #^, Jintao Huang^2, #^, Di Hu^2^, Jing Sun^1^, Zhe Zhang^1^, Zhihao Li^1^, Bin-yan Zhong^2,^ *, Ran Zhu^1,^ *, Guanglin Wang^1,^ *

^1^State Key Laboratory of Radiation Medicine and Protection, School of Radiation Medicine and Protection, Collaborative Innovation Center of Radiation Medicine of Jiangsu Higher Education Institutions, Soochow University, Suzhou 215123, China

^2^Department of Interventional Radiology, The First Affiliated Hospital of Soochow University, Suzhou 215006, China

***Corresponding author:**

E-mail: [byzhongir@sina.com](mailto:byzhongir@sina.com)

E-mail: zhuran@suda.edu.cn

E-mail: [glwang@suda.edu.cn](mailto:glwang@suda.edu.cn)

The grafting rate of BP was calculated by ^1^H-NMR, which was obtained from the intensity of the methylene group on BP compared to the intensity of the methyl group on HA (1).

$grafting rate=\frac{\left( a+e \right)/4}{d/3} \times100\%$ (1)


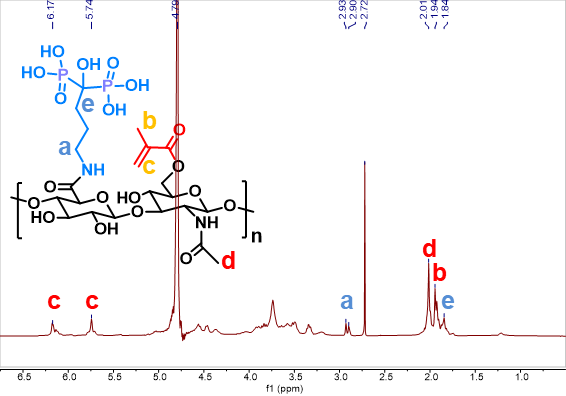


**Figure S1.** The ^1^H-NMR spectrum of HAMA-BP.


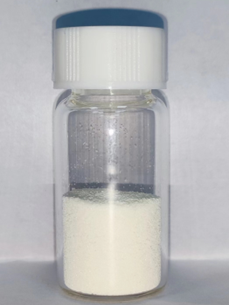


**Figure S2.** Photograph of HAMS powder.


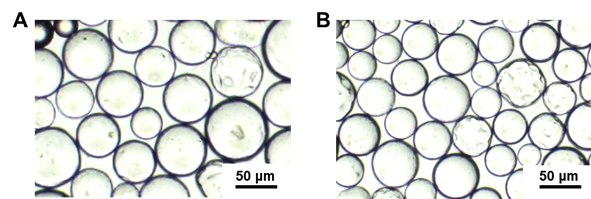

**Figure S3.** The optical images of the microspheres before (A) and after (B) radiolabeling.


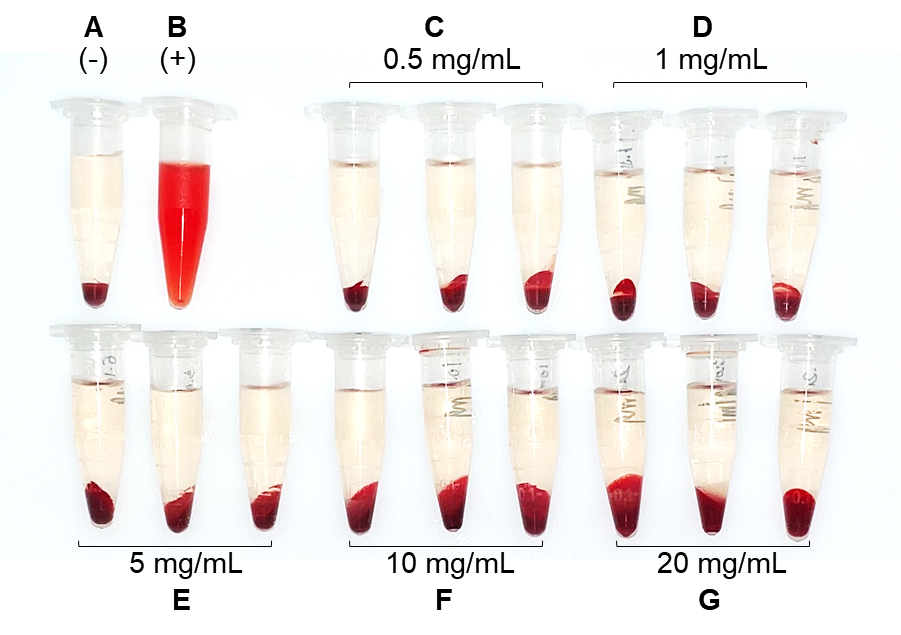


**Figure S4.** Appearance of microcentrifuge tubes for hemolysis experiments: (A) positive group, (B) control group, (C) 0.5 mg/mL sample group, (D) 1 mg/mL sample group, (E) 5 mg/mL sample group, (F) 10 mg/mL sample group, and (G) 20 mg/mL sample group, respectively.


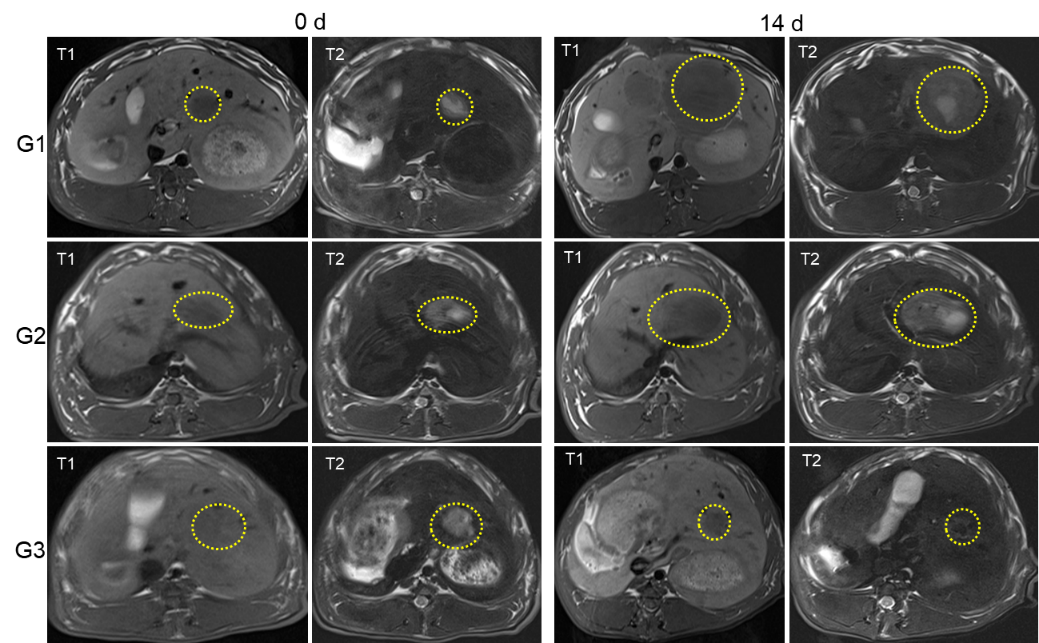


**Figure S5.** MRI images (T_1_WI and T_2_WI) of rabbits treated with different treatments (G1: saline, G2: HAMS, G3: ^90^Y-HAMS) by embolization administration. The tumors were highlighted with yellow dashed circles.
